# The role of priming in grammatical acceptability judgements for native versus non-native speakers: Effects of intelligibility

**DOI:** 10.1371/journal.pone.0275191

**Published:** 2022-09-28

**Authors:** Rodica R. Constantine, Douglas J. Getty, Scott H. Fraundorf

**Affiliations:** 1 Department of Psychology, University of Nevada, Las Vegas, Las Vegas, Nevada, United States of America; 2 Department of Psychology and Learning Research and Development Center, University of Pittsburgh, Pittsburgh, Pennsylvania, United States of America; Royal Holloway University of London, UNITED KINGDOM

## Abstract

Comprehenders frequently need to adapt to linguistic variability between talkers and dialects. Previous research has shown, given repeated exposure to quasi-grammatical structures, comprehenders begin to perceive them as more grammatical (Luka & Barsalou 2005, Luka & Choi 2012). We examined whether grammatical acceptability judgements differ for native versus non-native speech. In an exposure phase, native English speakers listened to, retyped, and rated the grammaticality of quasi-grammatical sentences (e.g., *What Emily is thankful for is that she is here*) spoken by a native or non-native speaker. In a subsequent test phase, participants rated additional sentences, some of which had the same structure as exposure sentences. Participants rated native-accented sentences as more grammatical, demonstrating a role for talker identity in perceptions of grammaticality. Furthermore, structures previously heard during the exposure phase were rated as more grammatical than novel unprimed structures, but only for the native speaker. Subset analyses suggest this effect is driven by speaker intelligibility, which holds implications for communication between native and non-native speakers.

## Introduction

Even within the same language, syntax can vary from one dialect or idiolect to another [1–9]. This variability could pose a challenge for comprehenders insofar as many theories of language processing propose comprehension partially relies on prior experience with the distribution of syntactic structures within language input [10–12]; thus, variability across dialects could render previous experience useless or even misleading. Nevertheless, previous research suggests that comprehenders can adapt to unfamiliar syntactic constructions [13–17].

The present study investigated whether the propensity for comprehenders to adapt to unfamiliar constructions is reduced when listening to a non-native versus a native speaker. We further contrast two theories that might give rise to such a reduction: (a) comprehenders’ expectation that grammatical incorrectness is more prevalent in non-native speech than in native speech or (b) non-native speech is less intelligible than native speech. One view of comprehenders’ adaptation to unfamiliar constructions holds that it is a form of implicit learning about the linguistic environment [13–15]. Under this account, participants may less readily adapt to syntactic structures of sentences spoken by a non-native speaker because they expect non-native speakers to make more syntactic errors [18, 19] and thereby be a less informative source about the linguistic environment than native speakers. On the other hand, if the reduced intelligibility of non-native speech is the driving force behind reduced adaptation to non-native speech [20, 21], we should expect equivalent adaptation to both speaker types once intelligibility is equalized.

### Structural priming and adaptation

The variability of syntax across dialects poses a challenge for comprehenders when they are prompted to interpret an unfamiliar structure. However, several experiments have provided evidence that people are able to adapt their syntactic processing to unfamiliar structures, despite having little to no prior experience with them.

First, past research tested the online processing of syntactic constructions specific to particular dialects, such as the *needs/wants/likes* + Past Participle (V-en) construction [3–5, 22], to find that readers unfamiliar with these constructions initially process them slowly but come to process them more quickly with repeated exposures [16, 17, 23], making their reading patterns more like native users of the construction [15].

Second, and most relevant to the present study, Luka and colleagues [13, 14] investigated the perceived grammaticality of novel or quasi-grammatical structures such as (1) and (2) below. In Luka and Choi [14], participants who were exposed to a new sentence construction (1) during an exposure phase would later judge a different sentence with the same structure (2) as more grammatical than when they lacked such exposure. Indeed, even a single exposure to a similar or identical sentence structure was sufficient to increase a participant’s perception of grammaticality. This effect persisted even after a 48-hour delay between initial exposure to new syntactic structures and a later test (Experiments 1 and 2). Moreover, an effect was still found after 7 days, with larger effects found for more novel sentence structures than common syntactic patterns (Experiment 3). While these quasi-grammatical structures are not dialectally conditioned, they represent plausible “less-than-perfect” structures that comprehenders may be likely to encounter, suggesting a broader role for syntactic adaptation.

1. The wizard acquired from the gnomes three small vials that contained herbal potions.
2. Mr. Stratacelli purchased from us a violin case that concealed automatic weapons.

The propensity of comprehenders to adapt to unfamiliar sentence structures may be related to the more general phenomenon of *structural priming*, which is the tendency of exposure to a particular syntactic structure to facilitate subsequent processing of similar structures [24–26]. At least two different mechanisms have been proposed to explain structural priming as it connects to syntactic adaptation [27]. One possibility is that structural priming reflects short-term, residual activation of a recently processed syntactic structure, making it more accessible and likely to be used again [28]. Another is that structural priming is a form of long-term, implicit learning about the type of syntactic structures one should expect to encounter in the linguistic environment [14, 27, 29]. Some evidence suggests that structural priming is influenced by non-native accent [30, 31]; however, the precise influence of accent on structural priming and adaptation apart from social factors such as speaker identity, intelligibility, and perceived speaker knowledge varies across studies.

### Processing non-native speech

Until recently, most studies of structural adaptation and structural priming—like those of spoken language comprehension in general—have focused on comprehension of native speech or text. Regardless, comprehenders often need to understand the speech of individuals who are not native speakers of their language [32]. Non-native speech differs from native speech in several systematic ways, including slowed speech rate [33], altered stress patterns [34], systematic phoneme deviations [35], varying degrees of proficiency, and is, most critically for our current purposes, more likely to contain anomalous syntax [20, 21].

Here, we examine whether adaptation to quasi-grammatical structures differs for native—as compared to non-native—speech. This comparison is of interest for two reasons. First, in some theories of language processing 4 [36–38], communication is effective and efficient in part because of interlocutors’ ability to rapidly align with (i.e., adapt to) each other. If comprehenders adapt differently to non-native speech or do not adapt at all, it could entail practical consequences for communication with non-native speakers, such that interlocutors would be unlikely to achieve linguistic alignment as easily [39, 40]; and would in turn communicative less effectively.

Second, shedding light on how non-native speech is processed can inform our understanding of language processing more generally. Existing accounts of structural priming make competing predictions on how it might differ for non-native speech. One view holds that it reflects short-term activation of structural representations [28], such that listeners that correctly comprehend the non-native speech would plausibly show equivalent structural priming because the same structural representations are ultimately being activated. A contrasting view [41] suggests that structural priming reflects a form of implicit learning about what syntactic structures should be expected in the linguistic environment. Given that non-native speech is both more error-prone and variable than native speech [34, 42], it is a less reliable source of information about which syntactic structures should be expected in the future. Therefore, if structural priming reflects implicit learning, comprehenders may show reduced adaptation (i.e., reduced learning) to non-native speech.

Prior evidence suggests comprehenders may process native and non-native syntax differently. Hanulíková, Alphen, Goch, & Weber [18] examined how speaker identity modulates processing of gender agreement errors using event-related potentials (ERPs). Specifically, they examined the P600 component, which often occurs in response to a syntactic violation, a non-preferred syntactic structure, or a complex syntactic structure [43, 44]. A P600 response was observed when gender-agreement violations were spoken by a native speaker, but not when the same violations were produced by a speaker with a non-native accent. This difference was hypothesized to be driven by comprehenders’ contrasting expectations for native versus non-native speaker grammaticality, wherein they expect native speakers will avoid making grammatical mistakes while non-native speakers will make syntactic mistakes more frequently. The P600 response seems to originate from a deviation of expectation established when listening to a native speaker make an uncommon grammatical mistake. Since comprehenders may expect more mistakes from non-native speakers, the P600 response might not be elicited because the speech does not deviate from their expectations. Similar results were obtained by Seifeldin, Cantor, Boland, & Brennan [45], suggesting speaker identity modulates expectations of syntactic structure. They focused on copula deletion, a feature of African American English (AAE) and not standard English [46]. When three speakers—an AAE speaker, a White Standard speaker, and an Indian-accented speaker—used sentences including copula deletion, the White Standard speaker elicited a P600 response, whereas the AAE and Indian speakers did not. This result shows that identifying a speaker with the use of non-standard speech leads to lowered expectations they will use standard syntax. Finally, Squires [47] showed that comprehenders can prime to standard and non-standard grammatical structures, with short-term syntactic priming depending solely on linguistic information, while longer-term lexical repetition hinges on both linguistic information and the sociolinguistic cues of the speaker.

### Present study

Our present study investigated differences in how listeners evaluate dialectal variants in grammar when attending to speech by a native versus non-native speaker using an adaptation of the paradigm introduced by Luka and Choi [14]. In an initial exposure phase, participants listened to and retyped quasi-grammatical sentences spoken by a native or a non-native speaker. In a later test phase, participants encountered new tokens of the primed constructions, as well as other (unprimed) unfamiliar constructions, and rated them for grammaticality.

Thus, unlike in conventional structural priming tasks, which measure priming using immediate language production or measured reading times (for review, see [25]), our task measures perceived grammaticality as assessed by grammaticality ratings. Since this may or may not reflect the same mechanism underlying structural priming, we will refer to any observed effect of previous exposure to structure as *an effect of priming*. Note that, when we refer to an effect of priming throughout this paper, we intend to use it to describe an empirical pattern and not a specific mechanistic claim about cognitive processing (unless otherwise specified).

Following the conventional logic of linguistic priming studies (e.g., [48]), our key comparison was how ratings on the target item in the test phase were affected by the experimental manipulation of the prior context—whether participants had encountered a previous (prime) token of the same structure or not. We predicted that overall, participants would rate constructions they had been previously exposed to as more grammatical than structures to which they had not previously been exposed, effectively showing an effect of priming (and conceptually replicating Luka & Choi [14]). Our critical—and novel—question was whether comprehenders would show a reduced or eliminated effect of structural priming for non-native speakers.

Further, if comprehenders prime less from non-native speakers because they regard non-native speakers as less informative about the linguistic environment (i.e., an implicit-learning account) we should see a difference in ratings of native vs. non-native talkers even when considering only accurately understood sentences. On the other hand, if non-native speech reduces the effect of priming because of diminished intelligibility, then the effect of priming should be equivalent once we condition on successful comprehension. Of course, these accounts are not mutually exclusive—intelligibility and implicit learning may each contribute to differences in the effect of priming.

## Method

### Participants

This research was approved by the University of Pittsburgh Institutional Review Board under the protocol number PRO17090487. Participants provided online consent on Qualtrics (Qualtrics, Provo, UT) before taking the study. Seventy-five participants were recruited from the undergraduate subject pool at the University of Pittsburgh and received partial course credit for their participation. After removing participants who were not native English speakers (*n* = 9) and who took excessive time on the filler task (*n* = 1), analyses were conducted on data from 65 participants. All participants were at least 18 years of age (35 female and 30 male) and native English speakers. A small number of trials (17 total) were lost from two participants due to a data recording error. No other trials were removed before analysis.

### Materials

This study consisted of 90 English sentences, provided by Dr. Barbara Luka at Binghamton University ([14]; see stimuli and data at https://osf.io/5693g/). The sentences were selected on the basis of the previous norming done by Bard College undergraduates, who rated their grammaticality on a 7-point Likert scale ranging from Ungrammatical to Very Grammatical. Sentences were selected to be quasi-grammatical (i.e., of moderate grammatical acceptability). To supplement the grammaticality norms of Luka and Choi [14] with data from our own participant population, we conducted norming with a sample of 40 University of Pittsburgh undergraduates who did not participate in the main experiment. Participants assigned grammaticality ratings to written sentences in a random order. We conducted this norming study on written sentences to obtain ratings of the sentences that were not influenced by characteristics of the speaker. The mean grammaticality rating was 4.75 (*SD* = 0.63), which verifies that these sentences are perceived as moderately grammatical.

Subsequently, we recorded a spoken version of each stimulus sentence. Sentences were read aloud and recorded exactly as written by both a native and a non-native speaker of English. At the time of recording, our native speaker was a 31-year-old female who spoke American English; our non-native speaker was a 51-year-old female who was a native speaker of Romanian and started learning American English at the age of 26. All 90 sentences were read by both speakers and the assignment of sentences to speakers was counter-balanced across four conditions. Each participant was randomly assigned to one of four experimental conditions (see Table 1).

**Table 1:** Experimental design.

Version 1	Version 2
*Exposure Phase*	*Exposure Phase*
Prime: A–Native	Prime: A–Non-Native
Prime: B–Non-Native	Prime: B–Native
*Test Phase*	*Test Phase*
Primed: A–Native	Primed: A–Non-Native
Primed: B–Non-Native	Primed: B–Native
Unprimed: C—Native	Unprimed: C—Non-Native
Unprimed: D—Non-Native	Unprimed: D—Native
Version 3	Version 4
*Exposure Phase*	*Exposure Phase*
Prime: C–Native	Prime: C–Non-native
Prime: D–Non-native	Prime: D–Native
*Test Phase*	*Test Phase*
Primed: C–Native	Primed: C–Non-native
Primed: D–Non-Native	Primed: D–Native
Unprimed: A–Native	Unprimed: A–Non-native
Unprimed: B–Non-Native	Unprimed: B–Native

### Design

Four experimental lists were created (Table 1). Each list included an Exposure phase and a Test phase. In the Exposure phase, participants listened to and rated 30 sentences of varying sentence structures and normative grammaticality. Of these sentences, 15 were spoken by the native speaker and 15 by the non-native speaker, presented in a blocked design. In the Test phase, participants rated a further 60 sentences for their perceived correctness (30 per speaker). For each speaker, these included 15 Primed and 15 Unprimed sentences. Sentences in the Primed condition shared the same grammatical structure as a single sentence from the Exposure sentence. Each yoked prime-target pair consisted of a different grammatical structure. Prime and target pairs contained no content words in common. Sentences in the Unprimed condition consisted of a heterogenous mix of grammatical structures that had not been encountered anywhere else in the experiment. Therefore, the only time participants were exposed to a repeated structure was between the Exposure and Primed conditions, and that repetition only occurred within speaker conditions. For example, if a participant heard the sentence “*Phil extended the injunction to Bruce”* in the Exposure phase, the yoked Primed sentence was “*The court conceded the claims to the plaintiff”* while there would be no Unprimed sentence with the same structure for that participant.

Across lists, we counterbalanced (a) the order of the native versus non-native speaker, (b) the assignment of sentences to speaker, and (c) the assignment of sentences to the primed versus unprimed condition (see Table 1). Thus, any differences between speakers and any differences between the Primed and Unprimed conditions were fully de-confounded from order of presentation and sentence content.

Our focus in this design was comparing ratings of the target sentences in the Test phase as a function of the experimental prime manipulation—whether or not there had been a corresponding yoked Prime in the Exposure phase.

### Procedure

The experiment was administered through the survey platform Qualtrics. Participants were instructed that the study was designed to increase our understanding of how individuals perceive variation in grammatical correctness. In total, the experiment consisted of three parts: 1) the Exposure phase, 2) a buffer task, and 3) the Test phase.

During the Exposure phase, participants listened to audio files of sentences spoken by either a native or non-native speaker of English. After listening to each audio file, participants re-typed the sentence they heard in a response box. This re-typing procedure was included both to ensure that participants listened to the Exposure sentences and, most critically, to acquire a measure of participants’ immediate comprehension, which allowed us to test one of our questions of interest: Are any potential differences in grammaticality ratings given to native versus non-native speakers driven by the intelligibility of the speech?

Immediately after listening to and retyping a sentence, participants indicated the perceived grammatical correctness of the sentence on the provided Likert scale, ranging from 1 (Ungrammatical) to 7 (Highly Grammatical). Participants were given two examples of how this rating system would apply to sentences shown during the study. One example was an ungrammatical sentence which participants were told deserves a rating of 1 (“*For that the world is flat to be widely figured is unthinkable”*), while the other example was a well-formed grammatical sentence which deserves a rating of 7 (“*Keep doing that and you’ll need glasses”*). Participants rated 30 sentences (15 from the native speaker and 15 from the non-native speaker).

Following the Exposure phase, a mental rotation task [49] was used as a buffer before the Test phase. This task consists of identifying the final rotational position of 48 different three-dimensional objects of a two-alternative forced choice task. On average, participants took approximately 13.6 minutes to complete the distractor task (SD = 17.47).

For the Test phase, participants rated the perceived grammatical correctness of 60 sentences, where 30 were spoken by the native speaker and 30 were spoken by the non-native speaker. Throughout the entire Test phase, participants were no longer required to retype the sentences.

Following the Test phase, participants answered five Likert-scale items probing their perception of each speaker (“How similar to you did you find the speaker?”, “What do you think the education level of this speaker is?”, “How intelligent did you perceive this speaker to be?”, “How friendly did you think this speaker was?”, “How well does this speaker know English?”). Each participant answered all five items for both speakers in the order presented above. These items primarily served as a manipulation check to verify that participants perceived the two speakers as differing in their level of English knowledge or fluency. Also, since the perception of a speaker on dimensions such as friendliness and similarity has been shown to moderate adaptation effects [50], we could also test whether any observed effect of exposure to structures can be uniquely attributed to language proficiency or additional differences between other talker attributes.

## Results

### Perception questionnaire

**Fig 1:**
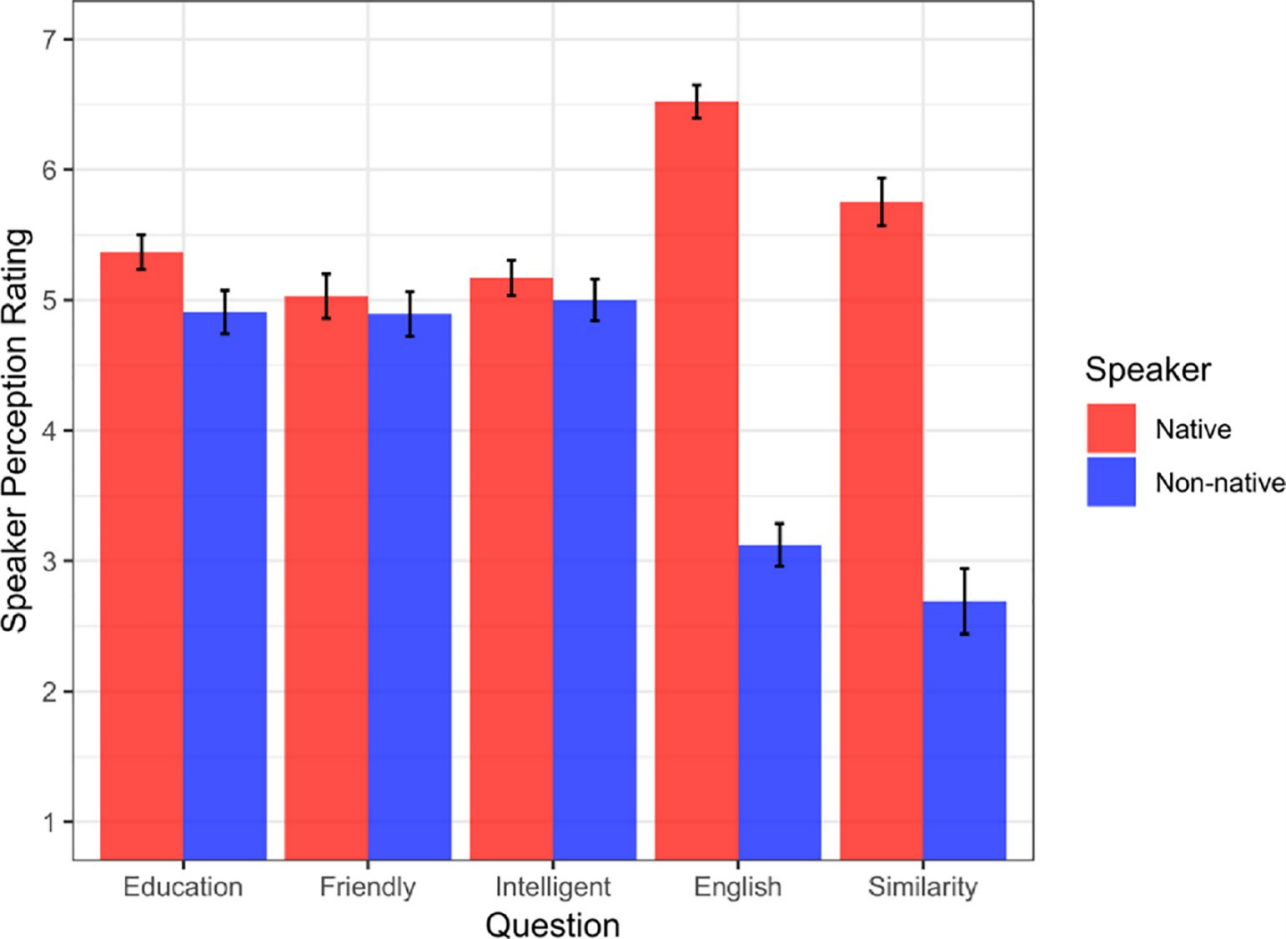
Mean perception ratings as a function of speaker type. Error bars represent standard error.

Within-subject paired-sample *t*-tests were administered for each of the five questions of the perception questionnaire (see Fig 1). The native speaker was rated significantly higher on the questions “How similar to you did you find this speaker?” (*t*(64) = 10.35, *p* < 0.0001), “What do you think the education level of this speaker is?” (*t*(64) = 2.48, *p* < 0.05), and especially “How well does this person know English?” (*t*(64) = 16.74, *p* < 0.0001). The speakers did not significantly differ on the questions, “How friendly did you perceive this speaker to be?” (*t*(64) = 0.63, *p* = 0.53), and “How intelligent did you find this speaker to be?”, (*t*(64) = 0.86, *p* = 0.39). Overall, these results indicate that our native-speaker participants perceived the non-native speaker as less knowledgeable of English, which supports the validity of our manipulation. (We examined whether any of these speaker perception ratings mediated the effect of speaker on grammaticality ratings and found no evidence for a mediational relationship.)

### Primary analysis

A linear mixed-effects regression analysis was conducted to examine how grammaticality ratings were influenced by the Speaker Type and Prime Type conditions. All data were analyzed in R using linear mixed effects regression models with the *lme4* [51] and *emmeans* packages [52]. In all models, the maximal random effects structure was initially tested as per [53] and then reduced by removing the least influential random effects until the model reached convergence.

The final model included fixed effects of Speaker Type [Native, Non-Native], Prime Type [Exposure, Primed, Unprimed], standardized normative grammaticality rating (from the norming study above), and an interaction term for Speaker Type x Prime Type. As a reminder, the Exposure phase occurred first and consisted of listening to primes from each speaker in turn. Then, in the Test phase, participants were exposed to Primed and Unprimed sentences from each speaker. Each of the Primed sentences were yoked to the specific structures that were heard in the Exposure phase, while the Unprimed structures were unique, only showing up in the Unprimed condition. We thus coded Prime Type using two orthogonal contrasts to test (a) differences between the Exposure phase and the Test phase (collapsed across Primed and Unprimed trials) in order to capture any effect of serial order on grammaticality ratings and (b) critically, difference between Primed versus Unprimed trials in the test phase in order to capture the effect of previous exposure to structures on grammaticality ratings (in other words, an effect of priming). Speaker Type was sum-coded. The random effects structure was near-maximal and included by-Subject and by-Item random intercepts, random subject slopes of Speaker Type and Prime Type, and random item slopes of Speaker Type.

**Fig 2:**
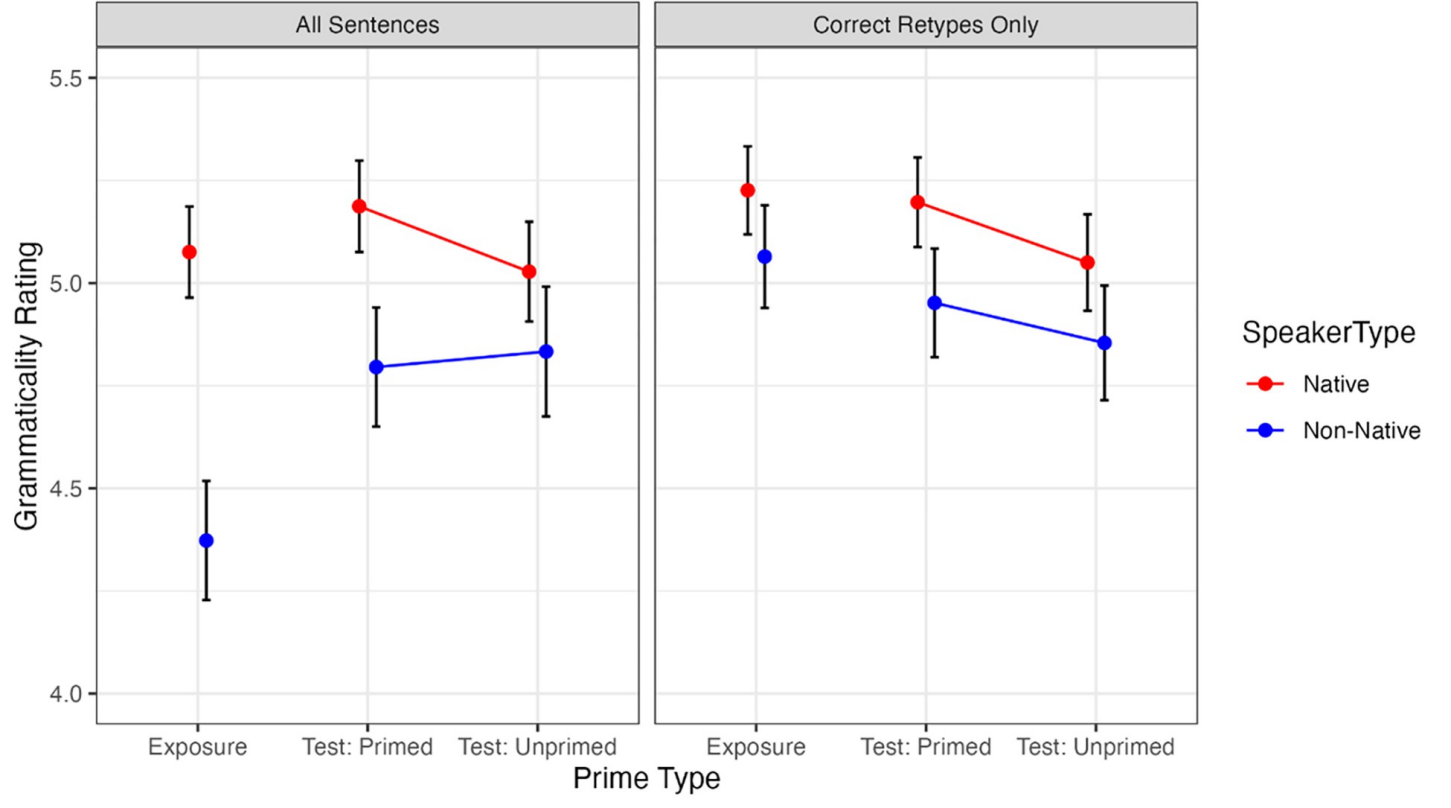
Mean grammaticality ratings (model-estimated) as a function of item type and speaker type. Error bars represent the standard error of the model-estimated marginal mean. Please note that because the means are model-estimated, values in the Unprimed condition are nearly, but not quite identical across both analyses (despite the underlying data being the same).

Table 2 presents the results of the linear mixed-effects model, and the first panel in Fig 2 presents the estimated marginal means. There was a main effect of Speaker Type, with the Native speaker rated higher on average than the Non-Native speaker (*B* = 0.42, *p* < 0.001), meaning that when participants heard sentences spoken by the Native English speaker, they perceived them as more grammatical overall, despite the Non-Native speaker having spoken the same sentences. There was a marginal increase in ratings between the Exposure and Test phases as evidenced by the Exposure versus Test contrast (*B =* 0.24, *p* = 0.05), and this effect significantly interacted with Speaker Type (*B* = -0.41, *p* < 0.05) such that this increase from Exposure to Test is larger for the Non-native speaker (see Fig 2).

**Table 2:** Linear mixed-effects regression results for model of all trials (N = 65, obs = 5833).

	*B*	SE	*t*	*p*
Intercept	4.88	0.09	50.65	< .001
Speaker Type	0.42	0.1	4.07	< .001
Exposure v. Test	0.24	0.12	1.95	0.052
Primed v. Unprimed	0.06	0.05	1.12	.26
Normed Rating	0.44	0.04	9.45	< .001
Speaker * Exposure v. Test	-0.41	0.16	-2.46	< .05
Speaker * Primed v. Unprimed	0.20	0.09	2.03	< .05

Our primary interest was in the effect of a previous exposure to set of structures on grammaticality ratings, which is encoded in our model as the Primed versus Unprimed contrast. The main effect of the Primed versus Unprimed contrast was not significant (*B* = 0.06, p = 0.26), indicating that, collapsing across Speaker Type conditions, an effect of previous exposure was not present. However, the interaction between Speaker Type and the Primed versus Unprimed contrast was significant (*B* = 0.20, *p* < 0.05), reflecting the fact that the difference between the Primed and Unprimed condition is larger in the Native speaker condition than the Non-Native speaker. In other words, the effect of previous structural exposure on grammaticality ratings appears to be contingent on Speaker Type, which is consistent with our hypothesis.

To further examine the difference in adaptation between speakers, we ran separate linear mixed effects models on subsets of the data that only included either speaker type. These models contained a fixed effect of Prime Type with the same contrasts described above. For the Native speaker model, we found a significant effect of Primed versus Unprimed trials (*B* = 0.16, p < 0.02) and no difference between the Exposure and Test phase (*B* = 0.04, *p* = 0.72). The Non-Native speaker model showed no difference between the Primed and Unprimed trials (*B* = -0.04, *p* = 0.59), but a significant difference between the Exposure and Test trials (*B* = 0.44, *p* < 0.01). Taken together, these results suggest that the effect of previous structural exposure only resulted in increased grammaticality ratings in the native speaker condition, consistent with our hypothesis. However, there is also evidence for more general adaptation to the non-native speaker.

### Comparison of exposure and test phases

Given our experimental design, the difference between Primed and Unprimed ratings is the most direct test of whether previous exposure to structures leads to increased grammaticality ratings of those structures. This conclusion holds because the test sentences were kept constant and the Primed and Unprimed conditions were manipulated (and counterbalanced with sentence identity across participants), making this a true experimental design.

Another comparison of interest, however, is between the grammaticality ratings from the Exposure to Primed conditions. Such an increase in ratings between the experimental phases would be consistent with a syntactic adaptation effect, given that one might expect participants should rate a structure as more grammatical upon hearing it a second time. To test for this second feature of syntactic adaptation, we ran linear mixed-effects models for each speaker (Native or Non-native) individually. The models contained a treatment-coded fixed effect of Prime Type referenced to the Primed condition and standardized normative grammaticality rating. Having the Primed condition as the reference level gives two contrasts: Primed vs. Unprimed (as in the previous analysis) and Primed vs. Exposure, such that negative effects for both contrasts indicate that the ratings of the Primed condition are higher. For the native speaker, the effect of Primed vs. Unprimed was significant (*B* = -0.16, *p* < 0.05), again demonstrating an effect of previous exposure to structure on grammaticality ratings. The Primed vs. Exposure contrast was negative (indicating an increase from Exposure to Primed) but not significant (*B* = -0.12, *p* = 0.27). For the Non-native speaker, the Primed vs. Unprimed contrast was not significant (*B* = 0.04, *p* = 0.60), but the Primed vs. Exposure contrast was significant (*B* = -0.43, *p* < 0.05).

These results might suggest that the initial Exposure phase led to increased grammaticality ratings of Primed sentences for the Non-native speaker only, and not for the Native speaker. However, unlike the comparison of Primed versus Unprimed items within the Test phase, the comparison between the Exposure and Test phases is—by necessity—affected by the confounding with serial position within the experiment. For example, participants’ sensitivity to grammaticality, or their overall grammaticality ratings, may vary as a function of fatigue or practice with the task. (One other limitation of this analysis is that we do not have retype accuracy for either of the Test phase conditions (Primed and Unprimed), so no sentences from these conditions can be removed on the basis of their retype accuracy. By removing Exposure phase sentences with inaccurate retypes, the ratings for this phase will necessarily be artificially inflated by removing difficult-to-understand sentences. Thus, any comparisons in this analysis between the relative grammaticality rating of Exposure phase sentences and Test phase sentences is not an apples-to-apples comparison.) Indeed, we see at least one example of this in that (as discussed above) ratings for the non-native speaker increase overall from exposure to test as the participants become accustomed to that speaker. Another recent study [54] found that structural priming effects can decrease over trial order for primes spoken by native speakers, while they may increase for primes spoken by non-native speakers. Although serial order changes in trial-to-trial structural priming are not perfectly analogous to changes in grammaticality ratings after an exposure phase, this provides further evidence for the influence of serial position.

### Intelligibility analysis

Our analyses above suggest that non-native speech resulted in less of an effect of priming for comprehenders than native speech. We earlier discussed two mechanisms that might account for this result: reduced intelligibility of non-native speech or reduced implicit learning of syntactic structure. To begin to tease apart these possibilities, we considered participants’ retyping of sentences during the exposure phase: If the difference in the effect of priming is a function of intelligibility, then we should see an equivalent effect between the native and non-native speakers when we remove the Exposure-Primed pairs where participants incorrectly retyped the Exposure sentences.

To test whether retype accuracy influences the difference in rating between the Primed and Unprimed conditions, two raters judged whether sentences in the exposure phase were retyped with the same structure as the input. Since our interest was in syntactic processing, and the prime-target pairs contained no content words in common anyway, retypes were coded as *correct* even if there were errors such as tense changes, spelling errors, omissions of adjectives, or content word swaps as long as these changes did not influence the underlying sentence structure. Some examples of retypes that were categorized as *correct* are shared here (with the original reported first, and the retype reported second): *Current inmates evidently dislike electro-shock therapy* → *Current inmates evidently dislike electric shock therapy*; *Brook thumbed the diary for secrets* → *Brook filmed the diary for secret*s. Retypes were coded as *incorrect* if they contained errors that resulted in a sentence with a different global syntactic structure. This included errors such as changes of verb classes (e.g., transitive to intransitive verbs), omitted quantifiers, and swapping of word classes (e.g., conjunction in place of a preposition. Some examples of incorrect retypes are shared here (again, the original reported first, and the retype reported second): *The critic yawned his disdain* → *The pretty swan is this day*; *Riley wants to receive Warsaw on his radio* → *Riley wants to receive water when he is radio*. Raters were blind to whether the sentence was in the Native or Non-Native condition. Inter-rater reliability was high (Cohen’s kappa = 0.94).

This coding yielded evidence that the Non-Native speaker was less intelligible: Participants correctly retyped 87% of the sentences from the Native speaker, but only 67% of the sentences from the Non-Native talker (*t* (64) = 9.80, p < .001). We attended to the question of whether this difference in intelligibility accounts for adaptation differences between Native and Non-Native speakers by conducting the same linear mixed-effects model as above, restricting the analysis to the subset of prime-target pairs (in the Exposure and Primed conditions) for which participants correctly retyped the primes in the Exposure phase.

Table 3 presents the results of this model. These results are also reported graphically in the second panel of Fig 2. The Primed versus Unprimed contrast was significant (*B* = 0.12, *p* < 0.05). The main effect of Speaker Type remained significant ($\hat{B}$ = 0.20, *p* < 0.05) such that participants perceived the Non-Native speaker as less grammatical overall, even when considering only trials on which participants reproduced the prime structure in the Exposure phase. (Since we did not ask participants to reproduce the test sentences during the *Test* phase, however, this set of trials may include trials on which participants did not correctly perceive the test sentence.).

**Table 3:** Linear mixed-effects regression results for model of correctly retyped items (N = 65, obs = 4854).

	$\hat{B}$	SE	*t*	*p*
Intercept	5.05	0.09	56.18	< .001
Speaker Type	0.20	0.09	2.12	< .05
Exposure v. Test	-0.13	0.10	-1.29	.20
Primed v. Unprimed	0.12	0.06	2.08	.04
Normed Rating	0.43	0.04	10.16	< .001
Speaker * Exposure v. Test	0.06	0.15	0.39	.70
Speaker * Primed v. Unprimed	0.05	0.10	0.47	0.64

Critically, when considering only Exposure sentences for which the participant correctly reproduced the structure, the interaction between Speaker Type and the Primed versus Unprimed contrast was no longer significant (*B* = 0.05, *p* = 0.64), suggesting that as long as the prime structures were faithfully understood, there was no longer a difference between the Native and Non-Native conditions in the magnitude of the effect of previous structural exposure. Rather, there was only a main effect of Primed versus Unprimed such that, overall, structures which had a previous exposure were rated as more grammatical than unprimed structures. In other words, we observe an interaction between Speaker Type and Primed versus Unprimed conditions when sentences with unintelligible primes are included, but when we remove sentences with unintelligible primes, we observe only a main effect of Priming across speakers.

It is possible that the absence of the interaction among correctly retyped primes simply reflects reduced statistical power in this subset of the data; however, power is unlikely to be the whole story because, when restricting to correct retypes, the parameter estimate for the critical interaction (i.e., its effect size) was also reduced fourfold from $\hat{B}$ = 0.20 to $\hat{B}$ = 0.05. That is, it was not the case that the effect remained of similar magnitude but simply became too difficult to reliably detect in the subset; rather, the effect itself greatly decreased. Thus, this finding demonstrates that intelligibility plays an influential role in the degree of influence that previous exposure to a structure has on native versus non-native speech.

### Normed vs. experimental ratings

Although the results presented above demonstrate that native and non-native speakers are given different grammaticality ratings, these data do not answer the question of how these ratings compare to a case where there the effect of speaker accent is unclear or not present, as in the case of written text. That is, does a native accent increase perceptions of grammaticality, does a non-native accent decrease it, or both? Fortunately, our normed ratings (which were collected on written stimuli) allow for such a comparison, and we thus ran an explicit comparison between the written norms and the ratings collected in the experiment.

We ran a by-item linear mixed-effects model on the Exposure phase data comparing the normed grammaticality ratings to the grammaticality ratings obtained from the experiment from each speaker. This resulted in a model with a single factor containing three levels: Normed, Native, and Non-native. We treatment-coded the conditions, with the normed condition as the reference level. Both contrasts were significant, with the Native contrast showing an increase in grammaticality ratings relative to the normed ratings (*B* = 0.32, *SE* = 0.11, *t* = 2.82, *p* < 0.001), and the Non-native contrast showing a decrease—of similar magnitude—relative to the normed ratings (*B* = -0.39, *SE* = 0.11, *t* = -3.46, *p* < 0.001). This indicates that a native speaker producing quasi-grammatical sentences leads to an increase in grammaticality ratings, while a non-native speaker producing the same sentences leads to a comparably sized decrease in ratings.

We ran an analogous model on the Test phase data (pooling across the Primed and Unprimed conditions). Only the Native contrast was significant (*B* = 0.35, *SE* = 0.08, *t* = 4.38, *p* < 0.001), consistent with an increase in grammaticality ratings for the native speaker relative to the normed ratings. The Non-native contrast was not significantly different than the normed ratings (*B* = 0.06, *SE* = 0.08, *t* = 0.75, *p* = 0.45). This suggests that, after an initial exposure period, listeners do not perceive the non-native speaker to be less grammatical than written norming sentences. Nevertheless, the native speaker producing quasi-grammatical sentences still leads to a small increase in grammaticality ratings, possibly indicating these quasi-grammatical structures obtain an endorsement from a speaker with clear native-like proficiency.

## Discussion

Participants listened to and rated the grammaticality of quasi-grammatical sentences spoken by native and non-native speakers across an initial Exposure phase and a subsequent Test phase. We found that participants rated the native speaker as more grammatical overall, even though the same sentences were read by the non-native speaker, which suggests that accent plays a role in the perception of grammatical acceptability.

Furthermore, previous exposure to a quasi-grammatical structure (the effect of priming) increased grammaticality ratings only when produced by a native speaker. There was no effect of previous exposure to structure when these sentences were produced by a non-native speaker.

We considered the possibility that this difference reflects comprehenders’ differing top-down expectations about a non-native talker, a difference in intelligibility, or both. The results suggest that this effect appeared to be driven entirely by the reduced intelligibility of the non-native speech: When considering only correctly retyped sentences in the Exposure phase, the magnitude of the effect of priming did not significantly differ between native and non-native speakers.

### Theoretical implications

Our study shows that perceived grammaticality may vary as a function of speaker identity in at least two ways. First, we found that accent indirectly affected the influence of previous exposure to quasi-grammatical structures. Overall, participants showed an effect of structural priming only for the native—and not the non-native—speaker, which appeared to be driven by the reduced intelligibility of non-native speech. This difference in intelligibility could potentially stem from factors such as variability of speaking rate, language proficiency, and accent familiarity [55–58]. These results, however, do not negate that in other cases syntactic adaptation may also be driven by expectations about ungrammaticalities in non-native speech [19].

We found a second influence of talker identity on perceived grammaticality wherein the non-native speaker was perceived as less grammatical than the native speaker overall. Unlike the previously discussed effects of priming of structure, the reduced perception of grammaticality of the non-native speaker does not appear to be driven by intelligibility since it was also obtained when only considering sentences participants could correctly retype. Instead, this finding supports the expectations account of language processing suggested by Hanulíková et al. [18], who showed using stimuli with overt grammatical errors that speaker identity modulates expectations of grammatical correctness. Specifically, the reduced P600 response when an error was produced by a non-native talker could be explained by comprehenders holding expectations that non-native speakers are more likely to make errors; thus, an error produced by a non-native speaker would less readily elicit a P600 response because it deviates less from comprehenders’ expectations.

In the present study, we tested a different class of stimuli: quasi-grammatical sentences, which did not include obvious grammatical errors. Nevertheless, the Hanuliková et al. [18] account could be extended to our results. Participants’ top-down expectation that the non-native speaker would make more errors may have led them to perceive those sentences as less grammatical. Indeed, other work has shown that even when intelligibility is controlled for, native speech is preferred to non-native speech by both native and non-native speakers [59, 60], that listeners only adjust their linguistic representations to accommodate for a newly introduced characteristic when it is spoken by a native as opposed to non-native speaker [19], and that listeners infer more syntactic errors in non-native utterances [61]. Together, these results indicate comprehenders attend to speaker identity, which in turn shapes their expectations of syntax based on their preconceived sociolinguistic notions of how a non-standard speaker may speak in general.

Nonetheless, the Hanuliková et al. [18] expectations account does not explain why the grammaticality ratings for the non-native speaker increased from the exposure to the test phase. The unfamiliarity of processing non-native accented speech [62] may account for comprehenders’ preference for native speech [20, 21, 63], as increased difficulty with processing fluency shares an inverse relationship with ratings of grammatical acceptability [64, 65]. Additionally, comprehenders may attribute this heightened processing difficulty to the non-native speaker’s ungrammaticality or lack of competence in speaking their native language [66–69]. Ultimately, this diminished negative effect of non-native accent might be explained by repeated exposure to the non-native speaker making processing fluency less cognitively demanding for comprehenders.

### Adaptation in production versus comprehension

We think it is striking that participants aligned no more, or even less, to non-native speakers’ grammatical structures, given that the opposite may occur in speech *production*. Work in speech production has shown that interlocutors align with each other at different linguistic levels (e.g., temporal phonetic, lexical, syntactic; [70, 71] and that this alignment is mediated by social factors, such as the perceptions both of one’s own language proficiency and one’s interlocutor [30, 72, 73], as well as how socially similar they are to one other [74]. This may reflect an explicit attempt to accommodate non-native speakers to achieve linguistic alignment while engaged in dialogue [75] and/or to serve social purposes, such as demonstrating liking of an interlocutor (see Communication Accommodation Theory: [76, 77]).

Nevertheless, even in speech production, there are limits to accommodation. Although native interlocutors may attempt to accommodate a non-native interlocutor, they do not adjust the implicit linguistic representations of their native language based on a non-native speaker’s linguistic input [19]. Furthermore, Ostrand & Ferreira [78] showed that speakers engage in linguistic alignment only to the extent that it enhances communicative utility, such that when communicative utility is controlled, speakers demonstrate syntactic alignment only for partner-general as opposed to partner-specific distributions of language input to account for the overall syntactic distributions of their linguistic context.

Why might speakers sometimes accommodate and align to non-native speakers in language production while not showing evidence of this in comprehension of structure in our experiment? One possible explanation focuses on the goals of task. When the goal is only to comprehend (which is true of the monologue scenario in our experiment), it makes sense to learn from information that is reliable and thus more likely to appear again the in the future, and likewise to discount unreliable information (as in Lev-Ari & Peperkamp [19]). Alternatively, when the goal is to comprehend and also be understood (particularly in dialogue), it is sensible to produce potentially non-standard or quasi-grammatical constructions if they are similar to ones that the non-native speaker may produce to better linguistically align (but, see Kim & Chamorro [74], for contradictory findings), even if producing these non-standard constructions does not reflect a change in the underlying linguistic representations.

### Methodological implications

Our results also have implications for how researchers can measure grammaticality. The fact that participants rated the sentences spoken by the non-native speaker as less grammatical than the native speaker—even though the text of the sentences was equated across lists—supports the claim that grammaticality ratings can be influenced by social factors, such as speaker identity (for review, see [65, 79]). When not controlled for, speaker identity could account for observed differences in putative grammaticality in multiple ways, with both a non-native accent decreasing perceived grammaticality, and a native accent lending credibility to quasi-grammatical structures and increasing their perceived grammaticality. It is important to control or systematically vary such factors.

### Limitations and future directions

An empirical limitation of the current data is that, while we found an effect in the Test phase as a function of the priming manipulation, we did not observe a difference in grammaticality ratings of the Primed sentences *between* the Exposure and Test phases for the native speaker. We might have expected such an effect if priming of the target structures resulted in a large increase in their perceived grammaticality from the first (Exposure) to second (Test) encounter. One critique, then, is that we do not show unambiguous evidence of syntactic adaptation. Perhaps the apparent effect of priming that we observed could be driven by a spurious decrease in ratings of novel (i.e., unprimed) structures from Exposure to Test. However, any comparison between the Exposure and Test phase is not a true experimental manipulation and necessarily confounded with serial position; the absence of a significant increase in ratings across phases could be driven by fatigue or general task adaptation. By comparison, when holding constant the target sentences in the Test phase and comparing the manipulation of the prior prime context—a true experimental manipulation—we do see a significant difference between Primed and Unprimed targets.

There are other limitations of this work that may present fruitful areas for future research. Understanding how a speaker’s accent may change perceptions of grammatical correctness prompts many new questions concerning differences in language perception, particularly those oriented around the effect different accents may have on the comprehender. For example, while we tested the effect of a non-native accent from a foreign language, perceptions of grammaticality might also differ when listening to a native talker with a different dialect form one’s own (e.g., AAE, British English). Alternatively, it remains unknown how one would perceive grammaticality given a non-native accent of a language that is more closely related to the target language (e.g., Dutch or German). Another variation to consider is whether a comprehender’s perceived grammaticality of a non-native accent changes as a function of a listener’s familiarity with that accent. A further direction would be to test whether participants’ adaptation to the native speakers’ syntactic structures is speaker-specific [80]. For instance, a different speaker could be used for the test phase to further assess whether adaptation generalizes across speakers.

Additionally, varying the participant pool (e.g., from native English speakers to non-native speakers) could reveal differences in perception as well as how cultural identity plays a role in expectations of native versus non-native speech. For example, Romanian-speaking participants may find other Romanian speakers to have more grammaticality when speaking English than would American English speakers. This could be due to having prior exposure to other Romanian speakers speaking in English, which might make their speech more intelligible. Moreover, Romanian-speaking participants likely have different expectations for a Romanian speaker’s grammatical deviations due to familiarity gained through past exposure, perhaps meaning violations of expectation would be less marked. Finally, Romanian-speaking participants may not hear a Romanian speaker’s grammaticality errors as starkly since they do not perceive such a discrepancy between the identity of the Romanian speaker and themselves.

Lastly, we used a meta-linguistic task wherein people rated the grammaticality of sentences, which is not what one typically does in naturalistic language processing. Using language processing tasks with higher ecological validity, such as communication-based tasks or online measures of grammatical processing, could be helpful in examining the influence of accent on syntactic processing.

## Conclusion

Participants rated sentences spoken by a native speaker as being more grammatical than those spoken by a non-native speaker despite both speakers producing the same set of sentences across counterbalanced lists. This finding indicates that existing expectations attributed to speaker identity actively modulate general perception of grammaticality. Participants also showed greater adaptation to syntactic structures spoken by the native speaker, which appeared to be driven by the greater intelligibility of native-accented speech. To the extent that structural adaptation may contribute to alignment and successful comprehension between interlocutors, this work reveals an additional communicative challenge in the context of linguistic variability.

## Supporting information

S1 Data (CSV)
